# Inhibition of MCF-7 breast cancer cell-induced platelet aggregation using a combination of antiplatelet drugs

**DOI:** 10.3892/ol.2012.1074

**Published:** 2012-12-13

**Authors:** LIAN LIAN, WEI LI, ZHEN-YU LI, YI-XIANG MAO, YOU-TAO ZHANG, YI-MING ZHAO, KAI CHEN, WEI-MING DUAN, MIN TAO

**Affiliations:** 1 Department of Oncology, The First Affiliated Hospital of Soochow University, Suzhou 215006;; 2 Department of Oncology, Suzhou Xiangcheng People’s Hospital, Suzhou 215131, P.R. China;; 3 Department of Molecular and Cellular Biochemistry, University of Kentucky, Lexington, KY 40536-0200, USA;; 4 Department of Clinical Laboratory, The First Affiliated Hospital of Soochow University, Suzhou 215006;; 5 Jiangsu Institute of Hematology, Key Laboratory of Thrombosis and Hemostasis, Ministry of Health, Suzhou 215006, P.R. China

**Keywords:** breast cancer, tumor cell-induced platelet aggregation, glycoprotein-Ib-IX, glycoprotein-IIb/IIIa, thromboxane A2, adenosine diphosphate

## Abstract

Cancer metastasis is a highly coordinated and dynamic multistep process in which cancer cells interact with a variety of host cells. Morphological studies have documented the association of circulating tumor cells with host platelets. Tumor cell-induced platelet aggregation (TCIPA) contributes significantly to hematogenous metastasis; however, the molecular mechanisms involved in breast cancer TCIPA are poorly characterized. In this study, MCF-7 metastatic human breast cancer cells induced dose-dependent aggregation of washed platelets. Four major platelet activation pathways, glycoprotein (GP)-Ib-IX, GPIIb/IIIa, thromboxane (TX)-A2 and adenosine diphosphate (ADP) were activated during TCIPA and were inhibited by their respective inhibitors, 7E3, SZ-1, aspirin and apyrase. Pretreatment of platelets with 7E3, SZ-1 or apyrase significantly inhibited TCIPA, while pretreatment with aspirin had no effect. Moreover, combined pretreatment of platelets with 7E3, SZ-1 and apyrase significantly inhibited TCIPA, compared to single inhibitors. Combinations of antiplatelet drugs may represent a promising strategy to prevent cancer metastasis.

## Introduction

Breast cancer is the most frequent cause of mortality in females in the developed world. Although early detection, precise resection using wide margins and systematic adjuvant therapy have improved survival, distant metastasis remains the leading cause of breast cancer-related mortality (1). Circulating tumor cells (CTCs) are isolated tumor cells that disseminate from the sites of metastatic and/or primary cancer, including breast cancer, and are identified and measured in peripheral blood (2). In a previous study, we observed that the rate of detection and number of CTCs correlated with the disease stage in breast cancer patients. Moreover, the assessment of CTCs in metastatic breast cancer patients predicts the efficacy of chemotherapy (1).

It is generally accepted that tumor cells become damaged during circulatory transport. This circulatory trauma may be due to humoral factors, including macrophages, natural killer cells and antibody-mediated complement lysis, as well as physical factors, including shear forces and mechanical trauma due to passage through the microvasculature. During hematogenous dissemination, CTCs undergo an extensive variety of interactions with host cells before they establish a secondary metastatic colony (3). The involvement of platelets in hematogenous metastasis has long been recognized. A correlation between venous thromboembolism and cancer was first observed by Trousseau in 1865 (4), while more recently, a study identified that the risk of cancer diagnosis is elevated following primary deep vein thrombosis (DVT) or pulmonary embolism (PE) (5). The ability of malignant tumor cells to aggregate platelets via tumor cell-induced platelet aggregation (TCIPA) (6,7) confers a number of advantages for the successful metastasis of cancer cells. When covered with a coat of platelets, a tumor cell acquires the ability to evade the body’s immune system. Indeed, platelets protect tumors from tumor necrosis factor α-mediated cytotoxicity (8). TCIPA also enables embolization of the large tumor-platelet aggregates at new extravasation sites within the microvasculature (9). Additionally, platelets facilitate the adhesion of tumor cells to the vascular endothelium (10) and release a number of growth factors which stimulate tumor cell growth (11). Furthermore, platelets contribute to tumor-induced angiogenesis by releasing angiogenic growth factors, including vascular endothelial growth factor (VEGF) (12).

In the initiation phase of primary hemostasis, interaction of the glycoprotein (GP) Ib/V/IX receptor complex with von Willebrand factor (vWF) on the surface of platelets mediates the adhesion of platelets. Subsequently, several platelet-activating factors, including thromboxane A2 (TXA2) and adenosine diphosphate (ADP), are secreted in an auto-crine/paracrine fashion and activate or prime approaching platelets. The release of ADP and TXA2 also leads to the conversion of the GPIIb/IIIa receptor into an active form, which mediates platelet aggregation. Tumor cells may use a variety of mechanisms to interact with platelets and induce platelet aggregation. Tumor cells express cell surface molecules, which induce tumor cell-platelet interactions and subsequent platelet aggregation (13), or induce platelet aggregation by generating typical platelet agonists, including thrombin (14) or ADP (15), which are considered as ‘soluble stimulators’ of platelet aggregation.

Characterization of the interactions between platelets and metastasizing tumor cells could potentially be used to develop therapies to disrupt this correlation and prevent cancer metastasis (16). Therefore, in the present study, we investigated the activation of the GPIb-IX, TXA2, ADP and GPIIb/IIIa pathways in platelets during TCIPA and determined the effect of inhibiting these pathways on TCIPA.

## Materials and methods

### Cell culture

MCF-7 human breast cancer cells (American Type Culture Collection, Manassas, VA, USA) were maintained in RPMI-1640 (Gibco, Grand Island, NY, USA) supplemented with 10% fetal calf serum (FCS; Hyclone Laboratories Inc., Logan, UT, USA), 100 U/ml penicillin and 100 mg/ml streptomycin at 37°C in a humidified atmosphere with 5% CO_2_ and passaged every 2–3 days to maintain exponential growth. The study was approved by the Ethics Committee of the First Affiliated Hospital of Soochow University, Suzhou, China.

### Reagents

Aspirin, apyrase and the anti-GPIb-IX complex monoclonal antibody, 7E3, were obtained from Sigma (St. Louis, MO, USA). The anti-GPIb-IX complex monoclonal antibody, SZ-1, was prepared according to our previously described methods (17). Fluorescein-isothiocyanate (FITC)-conjugated monoclonal antibody against high-affinity GPIIb/IIIa (PAC-1-FITC) was purchased from Becton Dickinson Biosciences (Mississauga, ON, Canada). Recombinant-phycoerythrin (PE)-conjugated monoclonal antibody against human platelet GPIb (CD42b-PE) was purchased from Dako Diagnostics (Glostrup, Denmark).

### Preparation of washed platelets

Fresh blood obtained from healthy volunteers was anticoagulated with a 1/7 volume of acid-citrate dextrose (ACD; 85 mM trisodium citrate, 110 mM dextrose, 78 mM citric acid) as previously described (18), washed twice with CGS buffer (0.12 M sodium chloride, 0.0129 M trisodium citrate and 0.03 M D-glucose; pH 6.5), resuspended in freshly prepared Tyrode’s buffer (18) and allowed to rest for at least 1 h at 37°C before use.

### Platelet aggregation

The interactions between platelets and tumor cells were measured by light aggregometry using a whole-blood ionized calcium lumi-aggregometer (Chrono-Log, Havertown, PA, USA). Briefly, 200 *μ*l platelets (200×10^6^ cells/ml) were placed in the aggregometer and incubated for 2 min at 37°C with stirring at 900 rpm, prior to the addition of cancer cells. TCIPA was initiated by the addition of 50 *μ*l tumor cells (0.05–50×10^6^ cells/ml) and the reactions were monitored and analyzed using the Aggro-link data processing system (Chrono-Log) for up to 15 min. Platelet aggregation was expressed as a percentage of the maximum aggregation rate. The structure of the platelet-tumor cell aggregates were also observed using an Olympus CKX41 phase-contrast microscope (Olympus, Melville, NY, USA).

### Flow cytometry analysis

The abundance of GPIb-IX and GPIIb/IIIa on the surface of the platelets during TCIPA was measured by flow cytometry. When TCIPA reached 50% maximal light transmission, the reaction was terminated by 10-fold dilution with physiological saline. The samples were then incubated with saturating concentrations (10 *μ*g/ml) of PE-anti GPIb-IX (CD42b-PE) or FITC-anti GPIIb/IIIa (CD41-FITC) in the dark for 5 min at room temperature and analyzed using a FC500 dual-laser five-color flow cytometer (Beckman Coulter, Fullerton, CA, USA). The mean fluorescence intensity was determined following correction for cell autofluorescence.

### Enzyme-linked immunosorbent assay (ELISA)

As TXA2 quickly transforms into TXB2 in aqueous solution, the concentration of TXB2 was measured using an enzyme immunoassay kit (Amersham Pharmacia Biotech, Buckinghamshire, UK) (18). When TCIPA reached 50% maximal light transmission, the reaction was terminated by 10-fold dilution with physiological saline, centrifuged and the supernatants were assayed for the generation of TXB2 using ELISA.

### ADP assays

ADP secreted from dense granules in stimulated platelets was measured using a whole blood ionized calcium lumi-aggregometer as previously described (19). Briefly, platelets were incubated with luciferin-luciferase reagent (440 U/ml luciferase and 4 *μ*g/ml luciferin) for 2 min at 37°C to convert ADP to adenosine triphosphate (ATP) and to generate chemiluminescence. Following incubation, the agonist was added and luminescence was monitored. To quantify the generation of ATP by platelets, standard curves were constructed using standard ATP.

### Statistical analysis

Each experiment was performed in triplicate, at least. Results are expressed as mean ± standard deviation (SD). Statistical analysis was performed using unpaired Student’s t-tests. P<0.05 was considered to indicate a statistically significant difference.

## Results

### MCF-7 cells induce platelet aggregation

**Figure 1.**
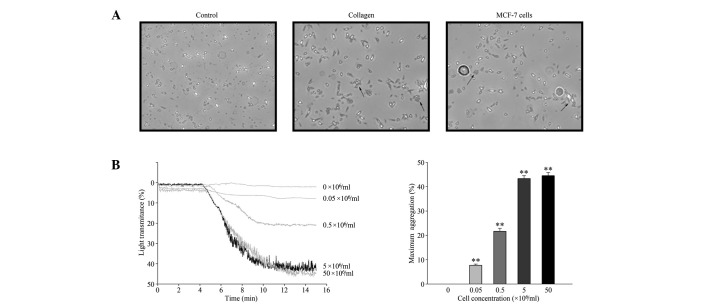
(A) Photomicrographs of MCF-7 cell-induced platelet aggregation. Untreated platelets were used as controls and collagen was used as a positive control to induce platelet aggregation. Platelet aggregation was triggered by MCF-7 cells at a concentration of 5×10^6^ cells/ml. (B) Quantitative measurement of MCF-7 cell-induced platelet aggregation. MCF-7 cells induced platelet aggregation in a concentration-dependent manner. ^**^P<0.01 vs. the control group.

The TCIPA effect of MCF-7 cells was observed using phase-contrast microscopy and quantified by light aggregometry. MCF-7 cells induced platelet aggregation in a similar manner to collagen, a classic inducer of platelet aggregation (Fig. 1A). MCF-7 cells induced platelet aggregation in a concentration-dependent manner (Fig. 1B), up to a maximal concentration of 5×10^6^ cells/ml. Therefore, 5×10^6^ cells/ml was selected as the standard cell concentration for all further experiments.

### Activation of the GPIb-IX, TXA2, ADP and GPIIb/IIIa pathways during TCIPA

**Figure 2.**
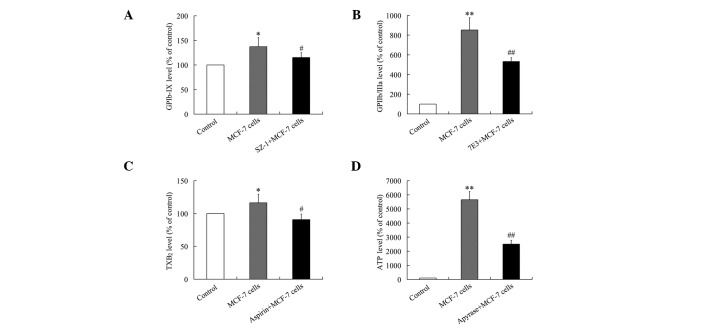
Activation of the GPIb-IX, TXA2, ADP and GPIIb/IIIa pathways during MCF-7 cell-induced TCIPA. GPIb-IX (A) and GPIIb/IIIa (B) were upregulated on the surface of platelets during TCIPA. These effects were attenuated by pretreatment of the platelets with SZ-1 or 7E3, respectively. (C) The stable metabolite of TXA2, TXB2, was upregulated during TCIPA. This effect was attenuated by pretreatment of the platelets with aspirin. (D) ADP release increased during TCIPA. This effect was attenuated by pretreatment of the platelets with apyrase. ^*^P<0.05 and ^**^P<0.01 vs. the control group; ^#^P<0.05 and ^##^P<0.01 vs. the MCF-7 cell-treated group. GP, glycoprotein; TX, thromboxane; ADP, adenosine diphosphate; TCIPA, tumor cell-induced platelet aggregation.

Activation of GPIb-IX and GPIIb/IIIa were evaluated by quantifying the abundance of GPIb-IX and GPIIb/IIIa on the surface of platelets using flow cytometry. As shown in Fig. 2A and B, GPIb-IX and GPIIb/IIIa were upregulated on the surface of platelets during TCIPA. These effects were repressed by pretreatment of the platelets with SZ-1 (10 *μ*g/ml) or 7E3 (20 *μ*g/ml), respectively.

TXA2 release was measured as the level of the stable TXA2 metabolite, TXB2, using ELISA (19). As shown in Fig. 2C, the increased levels of TXB2 observed during TCIPA were attenuated by pretreatment of the platelets with 50 *μ*g/ml aspirin.

ADP was measured as the level of ATP generated (20). As presented in Fig. 2D, the increased quantity of ADP released during TCIPA was inhibited by pretreatment of the platelets with 250 *μ*g/ml apyrase.

Thus, the GPIb-IX, TXA2, ADP and GPIIb/IIIa pathways were all activated during MCF-7-induced TCIPA and the activation of each pathway during TCIPA was repressed by pretreatment of the platelets with the appropriate inhibitors.

### Repression of TCIPA using a combination of GPIb-IX, ADP and/or GPIIb/IIIa pathway inhibitors

**Figure 3.**
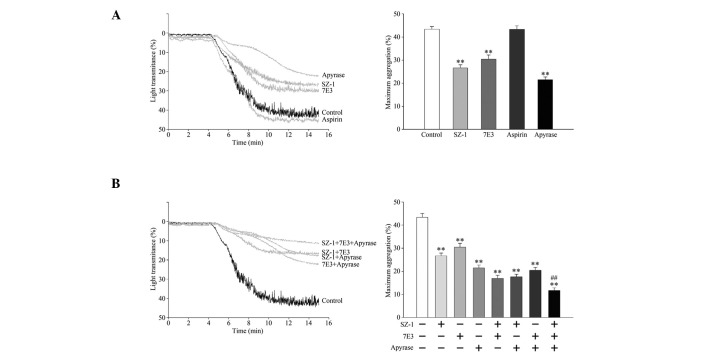
Effect of GPIb-IX, GPIIb/IIIa, TXA2 and/or ADP pathway inhibitors on MCF-7-induced platelet aggregation. (A) Platelets were pre-incubated for 5 min with SZ-1 (10 *μ*g/ml), 7E3 (20 *μ*g/ml), aspirin (50 *μ*g/ml) or apyrase (250 *μ*g/ml) before use in the MCF-7-induced platelet aggregation assays. (B) Inhibition of TCIPA using combinations of pathway inhibitors. ^**^P<0.01 vs. the control group; ^##^P<0.01 vs. all other groups. GP, glycoprotein; TX, thromboxane; ADP, adenosine diphosphate; TCIPA, tumor cell-induced platelet aggregation.

To investigate whether activation of the GPIb-IX, TXA2, ADP and GPIIb/IIIa pathways participated in MCF-7-induced TCIPA, platelets were pretreated with the inhibitors of each pathway. As shown in Fig. 3A and B, aspirin did not exert any significant effect on MCF-7-induced TCIPA. However, SZ-1, 7E3 and apyrase inhibited TCIPA, suggesting that MCF-7-induced TCIPA was executed through the GPIb-IX, ADP and GPIIb/IIIa pathways, but not the TXA2 pathway.

The combined effects of these platelet-aggregation inhibitors on MCF-7-induced TCIPA were investigated further. As shown in Fig. 3, paired pretreatment of platelets with SZ-1, 7E3 and/or apyrase lead to a slightly greater inhibition of MCF-7 cell-induced TCIPA and the combination of all three inhibitors significantly inhibited MCF-7-induced TCIPA, compared to the single inhibitors alone.

## Discussion

Metastasis is the major cause of mortality in breast cancer patients, yet there is no effective strategy to prevent tumor metastasis. Fewer than 0.01% of the cells that enter the bloodstream result in metastases (21). During hematogenous dissemination, the ability of circulating tumor cells to interact with platelets via TCIPA is believed to promote tumor cell survival within the circulation (22) and increase the arrest of tumor cell emboli within the microcirculation (21), thereby facilitating metastasis. TCIPA is currently gaining acceptance as a key intermediate step in the process of blood-borne metastasis (23). Pre-clinical animal models have demonstrated that pharmacologically- or genetically-induced thrombocytopenia (6,24) and platelet function defects are associated with reduced metastasis (24–26). These observations have prompted the use of antiplatelet and anticoagulation agents to prevent metastasis in experimental models (7,26) and human cancer patients (27). Thus, in the present study, we investigated the mechanisms involved in MCF-7 breast cancer cell-induced TCIPA and the effect of antiplatelet strategies on MCF-7-induced TCIPA.

In the present study, we observed that the GPIb-IX and GPIIb/IIIa pathways were activated during MCF-7-induced TCIPA and inhibition of the GPIb-IX and GPIIb/IIIa pathways repressed MCF-7-induced TCIPA. GPIb-IX and GPIIb/IIIa are the major platelet surface transmembrane receptors implicated in TCIPA (28). GPIb-IX mainly mediates platelet adhesion (29), while GPIIb/IIIa plays an important role in platelet aggregation (30). A previous study supported the hypothesis that the functions of GPIb-IX in platelets, which support normal hemostasis or pathological thrombosis also contribute to tumor malignancy (25). A functional absence of GPIb-IX correlated with a 15-fold reduction in the number of lung metastatic foci in an animal model using B16F10 melanoma cells, demonstrates that platelet GPIb-IX contributes to experimental metastasis (25). Competitive inhibition of platelet GPIIb/IIIa, either pharmacologically or by using antibodies against GPIIIa (31,32) and knockout of the GPIIIa subunit in mice also diminished the formation of metastases (33).

TXA2 (34) and ADP (35,36) are considered to be ‘soluble stimulators’ of platelet aggregation. In this study, we observed that the TXA2 and ADP pathways are activated during MCF-7 cell-induced TCIPA. Several lines of evidence support the hypothesis that TXA2 plays an important role in tumor metastasis. Firstly, TXA2 is a potent stimulator of platelet aggregation (37), which promotes the binding of tumor cell-platelet aggregates to endothelial cells (26). Secondly, several types of tumor cells release large amounts of TXA2, compared to normal tissues, which potentiates tumor growth *in vitro*(38). Thirdly, TXA2 has been demonstrated to increase endothelial cell migration and angiogenesis in several *in vitro* and *in vivo* models (39). Fourthly, TXA2 potentiates tumor cell growth in culture and increases metastasis in animals (34). Finally, the use of TXA2 inhibitors has been shown to reduce metastasis in animals (26,40). However, aspirin, an inhibitor of the TXA2-mediated pathway exerted no significant effect on MCF-7 cell-induced TCIPA, suggesting that the TXA2 aggregation pathway is not required during MCF-7-induced aggregation, in agreement with previous studies (5,24,41).

ADP is a potent pro-aggregatory agent, which is released during TCIPA induced by fibrosarcoma, breast carcinoma and neuroblastoma cells (36,42,43), whereas the ADP scavenger apyrase (35,42), ADP receptor antagonist ticlopidine (36) and ADP receptor inhibitor 2-methylthio-AMP (43) inhibit TCIPA. These findings are in agreement with the results of this study, in which MCF-7-induced TCIPA leads to activation of the ADP pathway and MCF-7-induced TCIPA is inhibited by the ADP scavenger, apyrase. ADP-mediated platelet activation is associated with a net increase in the release of VEGF in healthy individuals, with no effect on the release of endostatin. VEGF release in response to ADP-mediated platelet activation is abolished by selective inhibition of the P2Y12 receptor (44). Moreover, ADP depletion is associated with reduced formation of metastases *in vivo*(45) and improved biochemical control in prostate cancer patients receiving radiotherapy with curative intent (46).

We observed that combination of the GPIb-IX, GPIIb/IIIa and ADP pathway inhibitors exhibited a significant repression of TCIPA when compared with inhibition of a single pathway alone. Further studies are required to investigate the interactions between the GPIb-IX, GPIIb/IIIa and ADP pathways during TCIPA. The findings of the present study may be useful for the development of new clinical strategies to arrest TCIPA and prevent or reduce the formation of metastases.
